# Manifesto of the pediatricians of Emilia-Romagna region, Italy, in favor of vaccination against COVID in children 5–11 years old

**DOI:** 10.1186/s13052-022-01229-2

**Published:** 2022-03-05

**Authors:** Susanna Esposito, the Pediatricians of Emilia-Romagna Region, Italy

**Affiliations:** Pediatric Clinic, Pietro Barilla Children’s Hospital, University of Parma, Via Gramsci 14, 43126 Parma, Italy

**Keywords:** Children, COVID-19, COVID-19 vaccines, Paediatric infectious diseases, Caccine hesitancy

## Abstract

**Background:**

Following the authorization by the regulatory authorities of vaccination against COVID for children aged between 5 and 11, in Emilia-Romagna Region, Italy, the pediatricians of the Italian Society of Pediatrics (SIP), the Italian Society of Neonatology (SIN), the Cultural Association of Pediatrics, the Italian Federation of Pediatricians (FIMP) and the Italian Union of Family Pediatricians (SIMPeF), who work in the hospital and in the territorial setting, have made a univocal and convinced appeal in favor of vaccination also in this age group.

**Main findings:**

In order to contribute to a conscious choice, on the part of parents, based on exhaustive and correct information, a 24-point manifesto was developed. The manifesto showed that vaccines against COVID are the most effective and safest tool we have to counter the spread of SARS-CoV-2 and vaccination against COVID is a right of children just as it is for adults. Children between 5 and 11 years are not protected from the virus and a large part of the newly infected is this age. Although SARS-CoV-2 infection is certainly more benign in children, in some cases it can cause a serious pathology and long COVID. The stress caused by the pandemic, the prolonged closure of schools and the interruption of sports and recreational activities have had a devastating effect on the mental health of children and on the development of their personality. Vaccinating children against COVID serves to protect them from severe forms of disease and long COVID, allowing them to attend school face-to-face and lead a normal social life. The safety of vaccinatin is very high and vaccines against COVID have no influence on fertility nor can they cause developmental or growth side effects.

**Conclusions:**

The manifesto highlighted that the vaccine against COVID for children aged between 5 and 11 is effective and safe and represents an extraordinary gift for safeguarding health of the younger ones. The invitation, therefore, to parents is to have their children vaccinated against COVID as early as possible.

## Background

Following the authorization by the regulatory authorities of vaccination against COVID for children aged between 5 and 11 [1, 2], in Emilia-Romagna Region, Italy, the pediatricians of the Italian Society of Pediatrics (SIP), the Italian Society of Neonatology (SIN), the Cultural Association of Pediatrics, the Italian Federation of Pediatricians (FIMP) and the Italian union of family pediatricians (SIMPeF), who work in the hospital and in the territorial setting, have made a univocal and convinced appeal in favor of vaccination also in this age group, turning to parents so that they could understand the importance of vaccinating their children. The goal was to contribute to a conscious choice, on the part of parents, based on exhaustive and correct information through the creation of a 24-point manifesto.

## Main text

### Manifesto in favor of vaccination against COVID in children 5–11 years old

1. Vaccines against COVID are the most effective and safest tool we have to counter the spread of SARS-CoV-2 and vaccination against COVID is a right of children just as it is for adults [3].
2. Children between 5 and 11 years are not protected from the virus [4] and a large part of the newly infected is this age. The vaccine does not depress the child's ability to respond to infections but, on the contrary, allows the immune system to work "safely" by producing defense weapons in case of exposure to the virus [5].
3. Although SARS-CoV-2 infection is certainly more benign in children, in some cases it can cause a serious pathology such as multisystem inflammatory syndrome (MIS-C), which may also require hospitalization in intensive care [6, 7].
4. The long COVID, which consists in the persistence of pathological symptoms such as fatigue, difficulty concentrating, malaise 3 months after even mild SARS-CoV-2 infection, is a growing reality even in pediatric age and it can impact the child's overall physical and especially psychological well-being [8–11].
5. The stress caused by the pandemic, the prolonged closure of schools and the interruption of sports and recreational activities have had a devastating effect on the mental health of children and on the development of their personality [12] and, therefore, must be avoided by drastically breaking down with vaccination the circulation of the virus in all age groups, even in the pediatric age group [13].
6. Vaccinating children against COVID serves to protect them from severe forms of disease and long COVID, allowing them to attend school face-to-face and lead a normal social life [7].
7. While it has been shown that children with some chronic diseases are at greater risk of contracting the disease in a severe form, it is not possible to know which children, among those in good health, will have severe clinical manifestations, long COVID or psychosocial problems [10, 11].
8. The development of vaccines in children between 5 and 11 years old has not "skipped" any of the phases of verification of efficacy and safety [14]. The rapid development and approval is due to new technologies, the huge resources used and the commitment of the regulatory agencies. The number of children enrolled in the clinical trial that led to the authorization of the vaccine in children aged between 5 and 11 years is high, being a study on the pediatric population [14]. Millions of doses administered in various parts of the world (United States, Chile, Israel and Austria) are being added to this study, day after day, with no reports of adverse reactions that contraindicate its use also for these groups. age. In addition, none of the children who have been vaccinated have been hospi-talized for COVID so far [1, 2].
9. The pivotal study carried out has shown that vaccines on children between the ages of 5 and 11 are 91% effective in preventing symptomatic SARS-CoV-2 infection [14]. We also know from the data on adults that the vaccine's ability to prevent hospitalizations and deaths is much greater than its ability to prevent infection: therefore, vaccination can avoid all or almost all serious cases, including worrying ones [15].
10. The safety of vaccination, on over 3.5 million children vaccinated with one dose and 1 million with 2 doses in the USA, was very high: the most frequent side effects last a few hours and are pain at the injection site, headache, nausea and pain in muscles and joints [14].
11. In children between 5 and 11 years of age, vaccination against COVID does not seem to cause cardiac problems (myocarditis and pericarditis) [1, 2, 14], which have occurred very rarely in some children between 15 and 25 years and which have always resolved without problems [16]. Conversely, SARS-CoV-2 infection, like other viral diseases, can cause complications affecting the heart [6, 7].
12. Vaccinating children in this age group may mean fewer quarantines, less distance learning, fewer limits on extracurricular activities [17] and it will be possible to avoid swabs at every slightest symp-tom, with undoubted advantages also for the overall organization of the family.
13. The dose of antigen contained in the vaccine is 10 µg, one third of that given for ages 12 and up (30 µg) [1, 2]. Two intratramuscular injections are planned, three weeks apart [1, 2].
14. Vaccines against COVID are strongly recommended, due to the risk of serious COVID-related complications, on children with diseases such as immunodeficiencies, oncological diseases, heart disease, chronic kidney disease, chronic respiratory disease, severe obesity, non-type 1 diabetes. ade-quately controlled, trisomy 21 and neuromuscular pathologies [7].
15. There are no diseases for which there is an absolute contraindication to the mRNA vaccine against COVID [18]. Only in the case of a positive medical history for anaphylaxis, a careful medical history is advisable in order to evaluate whether to administer the vaccine by extending the post-vaccination observation time [17].
16. Vaccines against COVID have no influence on fertility nor can they cause developmental or growth side effects [19, 20].
17. Vaccinated children will also protect friends and relatives who come into contact with them and who, due to their health conditions (i.e., deficient immune defenses, underlying chronic diseases), are at risk of serious forms of disease [3].
18. The administration of vaccines against COVID can be concomitant or carried out at any time interval with other inactivated vaccines (i.e., anti-influenza, polio-diphtheria-tetanus-pertussis, anti-HPV). In the case of live attenuated virus vaccines (i.e., anti-measles-rubella-mumps-varicella) a minimum precautionary distance of 14 days must be maintained before or after administration of the COVID vaccine [21].
19. High vaccination coverage in children between 5 and 11 years will help reduce the circulation of SARS-CoV-2 and, consequently, the appearance in Europe of more contagious or aggressive viral variants that reduce the effectiveness of vaccines [22].
20. If you have had the infection (positive swab), a single dose of vaccine can be given within 6 months of infection [23]. Anyone with immunodeficiency will still have to receive two doses [23]. Evaluation of antibody titers is not useful in deciding whether to carry out vaccination [23]. If more than 6 months have passed since the infection it will be necessary to carry out two doses of vaccine [23]. This also applies to anyone who has had MIS-C.
21. In children with confirmed swab infection at least 15 days after the first dose, the second vaccination dose is not indicated [23]. Those who contract the infection after vaccination appear to have lower viral loads than the unvaccinated infected [24]. Partial vaccination and subsequent infection do not preclude a possible recall in the future.
22. In the last two years, COVID has absorbed a large part of health resources: getting vaccinated means contributing to the treatment of those suffering from other pathologies other than COVID and contributing to the regular resumption of treatment and prevention [25, 26].
23. Science does not allow us to predict the future but gives us clear indications about the present: we must have confidence, the choice of the vaccine even for 5–11 year old children is the most appro-priate for their health and to reiterate with enforce their rights.
24. For any doubts or concerns about the efficacy, safety and importance of vaccines for COVID, we invite you to consult your pediatrician or the staff of the local vaccination center, without waiting.

## Conclusions

The manifesto of the SIP, SIN, ACP, FIMP and SIMPeF pediatricians from Emilia-Romagna Region, Italy, highlighted that the vaccine against COVID for children aged between 5 and 11 is effective and safe and represents an extraordinary gift for safeguarding health of the younger ones. The invitation, therefore, to parents is to have their children vaccinated against COVID because as early as possible.

## Data Availability

All data generated during this study are included in the published article.

## Abbreviations

ACP: Cultural Association of Pediatrics
FIMP: Italian Federation of Pediatricians
MIS-C: Multisystem inflammatory syndrome in children
SIMPeF: Italian Union of Family Pediatricians
SIN: The Italian Society of Neonatology
SIP: Italian Society of Pediatrics
